# Supplementary material for: Iterative improvement in the automatic modular design of robot swarms
Source: PeerJ Comput Sci. 2020 Dec 7;6:e322. doi: 10.7717/peerj-cs.322 (PMC7924708; doi:10.7717/peerj-cs.322)
Supplement: Supplemental Information 3 [file peerj-cs-06-322-s003.zip › argos3/doc/api/standalone/a00500_source.html]

ARGoS: plugins/robots/generic/control\_interface/ci\_leds\_actuator.cpp Source File


- Main Page
- Related Pages
- Namespaces
- Classes
- Files

- File List
- File Members

# plugins/robots/generic/control\_interface/ci\_leds\_actuator.cpp

Go to the documentation of this file.

```
00001 
00008 #include "ci_leds_actuator.h"
00009 
00010 #ifdef ARGOS_WITH_LUA
00011 #include <argos3/core/wrappers/lua/lua_utility.h>
00012 #endif
00013 
00014 namespace argos {
00015 
00016    /****************************************/
00017    /****************************************/
00018 
00019 #ifdef ARGOS_WITH_LUA
00020    /*
00021     * This function expects the stack to have either two or four arguments.
00022     * The first argument must always be the index of the LED to set.
00023     * Then, in case two arguments are passed, the second argument can be the string
00024     * definition of a color. In case of four arguments, the RGB values are expected.
00025     */
00026    int LuaLEDSetSingleColor(lua_State* pt_lua_state) {
00027       /* Check parameters */
00028       if(lua_gettop(pt_lua_state) != 2 && lua_gettop(pt_lua_state) != 4) {
00029          return luaL_error(pt_lua_state, "robot.leds.set_single_color() expects 2 or 4 arguments");
00030       }
00031       luaL_checktype(pt_lua_state, 1, LUA_TNUMBER);
00032       size_t unIdx = lua_tonumber(pt_lua_state, 1);
00033       /* Get reference to actuator */
00034       CCI_LEDsActuator* pcAct = CLuaUtility::GetDeviceInstance<CCI_LEDsActuator>(pt_lua_state, "leds");
00035       if(unIdx < 1 || unIdx > pcAct->GetNumLEDs()) {
00036          return luaL_error(pt_lua_state, "passed index %d out of bounds [1,%d]", unIdx, pcAct->GetNumLEDs());
00037       }
00038       /* Create color buffer */
00039       CColor cColor;
00040       if(lua_gettop(pt_lua_state) == 2) {
00041          luaL_checktype(pt_lua_state, 2, LUA_TSTRING);
00042          try {
00043             cColor.Set(lua_tostring(pt_lua_state, 2));
00044          }
00045          catch(CARGoSException& ex) {
00046             return luaL_error(pt_lua_state, ex.what());
00047          }
00048       }
00049       else {
00050          luaL_checktype(pt_lua_state, 2, LUA_TNUMBER);
00051          luaL_checktype(pt_lua_state, 3, LUA_TNUMBER);
00052          luaL_checktype(pt_lua_state, 4, LUA_TNUMBER);
00053          cColor.Set(lua_tonumber(pt_lua_state, 2),
00054                     lua_tonumber(pt_lua_state, 3),
00055                     lua_tonumber(pt_lua_state, 4));
00056       }
00057       /* Perform action */
00058       pcAct->SetSingleColor(unIdx - 1, cColor);
00059       return 0;
00060    }
00061 
00062    /*
00063     * This function expects the stack to have either one or three arguments.
00064     * In case one argument is passed, it must be the string definition of a color.
00065     * In case of three arguments, the RGB values are expected.
00066     */
00067    int LuaLEDSetAllColors(lua_State* pt_lua_state) {
00068       /* Check parameters */
00069       if(lua_gettop(pt_lua_state) != 1 && lua_gettop(pt_lua_state) != 3) {
00070          return luaL_error(pt_lua_state, "robot.leds.set_all_colors() expects 1 or 3 arguments");
00071       }
00072       /* Create color buffer */
00073       CColor cColor;
00074       if(lua_gettop(pt_lua_state) == 1) {
00075          luaL_checktype(pt_lua_state, 1, LUA_TSTRING);
00076          try {
00077             cColor.Set(lua_tostring(pt_lua_state, 1));
00078          }
00079          catch(CARGoSException& ex) {
00080             return luaL_error(pt_lua_state, ex.what());
00081          }
00082       }
00083       else {
00084          luaL_checktype(pt_lua_state, 1, LUA_TNUMBER);
00085          luaL_checktype(pt_lua_state, 2, LUA_TNUMBER);
00086          luaL_checktype(pt_lua_state, 3, LUA_TNUMBER);
00087          cColor.Set(lua_tonumber(pt_lua_state, 1),
00088                     lua_tonumber(pt_lua_state, 2),
00089                     lua_tonumber(pt_lua_state, 3));
00090       }
00091       /* Perform action */
00092       CLuaUtility::GetDeviceInstance<CCI_LEDsActuator>(pt_lua_state, "leds")->
00093          SetAllColors(cColor);
00094       return 0;
00095    }
00096 #endif
00097 
00098    /****************************************/
00099    /****************************************/
00100 
00101    size_t CCI_LEDsActuator::GetNumLEDs() const {
00102      return m_tSettings.size();
00103    }
00104 
00105    /****************************************/
00106    /****************************************/
00107 
00108    void CCI_LEDsActuator::SetSingleColor(UInt32 un_led_number,
00109                                          const CColor& c_color) {
00110       m_tSettings[un_led_number] = c_color;
00111    }
00112       
00113    /****************************************/
00114    /****************************************/
00115 
00116    void CCI_LEDsActuator::SetAllColors(const CColor& c_color) {
00117       for(size_t i = 0; i < m_tSettings.size(); ++i) {
00118          m_tSettings[i] = c_color;
00119       }
00120    }
00121 
00122    /****************************************/
00123    /****************************************/
00124 
00125    void CCI_LEDsActuator::SetAllColors(const TSettings& c_colors) {
00126       m_tSettings = c_colors;
00127    }
00128 
00129    /****************************************/
00130    /****************************************/
00131 
00132    void CCI_LEDsActuator::SetSingleIntensity(UInt32 un_led_number,
00133                                              UInt8 un_intensity) {
00134       m_tSettings[un_led_number].SetAlpha(un_intensity);
00135    }
00136 
00137    /****************************************/
00138    /****************************************/
00139 
00140    void CCI_LEDsActuator::SetAllIntensities(UInt8 un_intensity) {
00141       for(size_t i = 0; i < m_tSettings.size(); ++i) {
00142          m_tSettings[i].SetAlpha(un_intensity);
00143       }
00144    }
00145 
00146    /****************************************/
00147    /****************************************/
00148 
00149 #ifdef ARGOS_WITH_LUA
00150    void CCI_LEDsActuator::CreateLuaState(lua_State* pt_lua_state) {
00151       CLuaUtility::OpenRobotStateTable(pt_lua_state, "leds");
00152       CLuaUtility::AddToTable(pt_lua_state, "_instance", this);
00153       CLuaUtility::AddToTable(pt_lua_state, "set_single_color", &LuaLEDSetSingleColor);
00154       CLuaUtility::AddToTable(pt_lua_state, "set_all_colors", &LuaLEDSetAllColors);
00155       CLuaUtility::CloseRobotStateTable(pt_lua_state);
00156    }
00157 #endif
00158 
00159    /****************************************/
00160    /****************************************/
00161 
00162 }
```

---

Generated on 10 Jul 2018 for ARGoS by 
 1.6.1
